# Cold Water Immersion or Foam Rolling? A Randomized Crossover Trial Comparing Recovery Effects on High-Intensity Performance and Markers of Muscle Damage and Inflammation in Soccer Players

**DOI:** 10.3390/sports14080328

**Published:** 2026-08-03

**Authors:** Balsam Messai, Slaheddine Delleli, Khaled Trabelsi, Achraf Ammar, Medina Srem Sai, John Elvis Hagan, Vlad Adrian Geantă, Hamdi Chtourou

**Affiliations:** 1 Higher Institute of Sport and Physical Education of Sfax, University of Sfax, Sfax 3000, Tunisia; messaibalssam@gmail.com (B.M.); sdelleli2018@gmail.com (S.D.); trabelsikhaled@gmail.com (K.T.); hamdi.chtourou@isseps.usf.tn (H.C.); 2 Physical Activity, Sport, and Health, UR18JS01, National Observatory of Sport, Tunis 1003, Tunisia; 3 Research Laboratory Education, Motricité, Sport et Santé, EM2S, LR19JS01, High Institute of Sport and Physical Education of Sfax, University of Sfax, Sfax 3000, Tunisia; 4 Department of Movement Sciences and Sports Training, School of Sport Science, The University of Jordan, Amman 11942, Jordan; 5 Department of Training and Movement Science, Institute of Sport Science, Johannes Gutenberg-University Mainz, 55122 Mainz, Germany; acammar@uni-mainz.de; 6 Research Laboratory, Molecular Bases of Human Pathology, LR19ES13, Faculty of Medicine of Sfax, University of Sfax, Sfax 3000, Tunisia; 7 Department of Nutrition and Food Technology, School of Agriculture, The University of Jordan, Amman 11942, Jordan; 8 Department of Health, Physical Education, Recreation and Sports, University of Education, Winneba P.O. Box 25, Ghana; mssai@uew.edu.gh; 9 Department of Health, Physical Education and Recreation, University of Cape Coast, Cape Coast PMB TF0494, Ghana; 10 Neurocognition and Action—Biomechanics Research Group, Faculty of Psychology and Sports Science, Bielefeld University, 33615 Bielefeld, Germany; 11 Department of Physical Education and Sport, Faculty of Physical Education and Sport, Aurel Vlaicu University of Arad, 310032 Arad, Romania

**Keywords:** cryotherapy, fatigue management, high-intensity intermittent exercise, myofascial release, muscle damage, perceptual responses

## Abstract

Background: Recovery between repeated high-intensity efforts is essential to limit fatigue and performance decline in soccer. This study examined the effects of cold water immersion (CWI), foam rolling (FR), and passive recovery during a 15 min interval between two 5 m shuttle run tests (5mSRTs) on physical performance, perceptual responses, and biochemical markers of muscle damage and inflammation. Methods: Fifteen soccer players (age: 22.6 ± 2.03 years) completed passive recovery, FR, and lower-body CWI at 15 °C in a randomized crossover design. Creatine kinase (CK), lactate dehydrogenase (LDH), C-reactive protein (CRP), aspartate transaminase (AST), delayed onset muscle soreness (DOMS), and belief questionnaire scores were assessed before the first 5mSRT and immediately after, and then 24 h, 48 h, and 72 h after the second 5mSRT. Results: Between the first and the second 5mSRT, TD remained unchanged following CWI (*p =* 0.21), whereas significant decreases were observed following passive recovery (*p =* 0.025) and FR (*p =* 0.028). Also, BD decreased after passive recovery (*p =* 0.03). At 48 h, CWI elicited lower CK (*p =* 0.01) and LDH (*p =* 0.003) than passive recovery, whereas FR resulted in lower AST concentrations (*p =* 0.01). Participants reported higher belief scores following CWI and FR than passive recovery throughout the recovery period (*p* < 0.05). No between-condition differences were observed for FI, PD, RPE, DOMS, or CRP (all *p* > 0.05). Conclusions: CWI may help preserve repeated-running performance and attenuate selected markers of muscle damage following repeated high-intensity intermittent exercise in soccer players, whereas FR demonstrated only limited physiological effects.

## 1. Introduction

Intermittent team sports (e.g., soccer) require athletes to repeatedly perform high-intensity efforts interspersed with brief recovery periods, making the ability to recover rapidly a key determinant of performance [1]. Following match play or simulated competition, athletes typically exhibit impairments in neuromuscular performance, increased fatigue perception and biochemical markers of muscle damage [2,3]. These alterations may persist for 48 to 72 h depending on participants’ characteristics and physical conditioning [2,4].

Facilitating the transition from exercise-induced stress and fatigue to a recovered state is essential for optimizing performance across most sports disciplines [5]. Accordingly, coaches and athletes continue to seek effective strategies capable of accelerating post-exercise recovery [5,6]. Among the available approaches, cooling interventions and self-myofascial release techniques have attracted substantial interest because of their practical implementation and potential benefits for recovery [6,7,8,9].

Cold water immersion (CWI) is one of the most commonly employed recovery methods with athletes [7,10,11]. In soccer, a previous study indicated that up to 90% of team sports used CWI as a core recovery strategy [12]. CWI can attenuate inflammatory responses and muscle soreness following high-intensity exercise [1,13,14]. Several physiological mechanisms may explain these effects, including reductions in muscle temperature, analgesic responses, peripheral vasoconstriction, edema formation, and tissue metabolism, which may attenuate muscle damage and inflammation [15,16]. However, the impact of CWI on performance recovery remains inconsistent. For example, CWI applied during halftime has been reported to have limited effects on subsequent soccer performance [17]. Practical recommendations emphasize that the effectiveness of water immersion strategies depends strongly on their application parameters such as temperature, duration, and immersion depth [18,19,20]. These factors, in addition to user characteristics and exercise modality, have been suggested to explain the discrepancy across studies [20,21].

Within the emerging myofascial literature, foam rolling (FR) has emerged as a practical and accessible recovery strategy [3,9]. Evidence suggests that FR is effective in improving recovery following exercise-induced muscle damage [22,23] and preventing performance decline [24,25]. FR has been shown to preserve or enhance neuromuscular performance and is particularly effective when applied in sport-specific contexts, such as during halftime, where it can attenuate performance decrements [3]. Additionally, a body of evidence has shown the effectiveness of FR in improving sprint, agility, jump performance, and muscle activation, as well as passive and dynamic range of motion [24,26]. Although their relative contribution remains uncertain, several mechanisms may explain the reported benefits of FR, including enhanced local blood flow, increased lactate clearance, modulation of pain perception, and changes in neuromuscular function [8,22].

Despite their widespread use, the comparative efficacy of these recovery modalities remains unclear. While some comparative studies in team sports athletes reported that FR elicited greater benefits on perceived fatigue and recovery markers following match-play [8,27], others indicated controversial results [1,28]. However, the effectiveness of each recovery strategy may differ according to the specific outcome evaluated [29].

Limited studies have examined CWI or FR strategies during halftime on soccer players’ subsequent performance and recovery [3,10,17], with inconsistent findings regarding each recovery method benefits. Furthermore, few studies have compared both strategies between two high-intensity repeated efforts separated by 15 min of recovery (i.e., that could simulate a competition) [8,27,28] on team sports players’ performance and muscle damage and inflammation biomarkers. However, these aforementioned studies’ findings were restricted to the effectiveness of the two recovery methods only at post-match. Based on the fact that during a competition setting, second-half performance may decrease and fatigue levels may increase [30], the present study compared CWI, FR and passive recovery methods during a 15 min recovery period between two 5 m shuttle run tests (5mSRT: 6 × 30 s with 35 s recovery in-between) on performance as well as perceived fatigue and biochemical markers of muscle damage and inflammation (before the first 5mSRT and after, and 24 h, 48 h, and 72 h after the second 5mSRT) [31,32]. The choice of the 5mSRT was in regard to the physical and physiological demands during its repeated efforts, which mimic those of a soccer match [31,32] despite the limited reflection of real technical–tactical skills [32]. Given the limited and inconsistent evidence comparing CWI and FR during short between-bout recovery periods, this study aimed to compare the effects of CWI, FR, and passive recovery applied during a 15 min interval between two 5mSRTs on repeated-effort performance and perceived fatigue, as well as on biochemical markers of muscle damage and inflammation assessed before the first 5mSRT, immediately after the second 5mSRT, and 24, 48, and 72 h thereafter. Because CWI and FR may influence recovery through distinct physiological and perceptual mechanisms, we adopted an exploratory approach and hypothesized that their effects would vary according to the outcome assessed and the timing of measurement.

## 2. Materials and Methods

### 2.1. Participants

An *a priori* power analysis was conducted using G*Power (version 3.1.9.6; University of Kiel, Kiel, Germany) with an alpha level of 0.05 and a power (1 − β) of 0.80. Based on a reported effect size of *f* = 0.39 for the impact of CWI on creatine kinase (CK) levels, derived from the summary effect size and 95% prediction interval [−2.23 to 0.69] [30], a repeated-measures ANOVA (within-factors) design was selected. During subsequent data screening, several outcome variables, including CK, deviated significantly from normality, necessitating the adoption of a rank-based, non-parametric primary analysis for those metrics. To evaluate whether the planned sample size remained statistically appropriate for these rank-based procedures, we utilized the asymptotic relative efficiency (ARE) as a supplementary sensitivity assessment. Specifically, for repeated measures across the k = 3 conditions, the operational efficiency factor was evaluated using ARE = 0.955 × k/(k + 1), and the required non-parametric sample size as N_n*p =*_ N_p_ × ARE [33]. Multiplying the parametric baseline by this design efficiency factor yields an adjusted non-parametric target threshold of N_n*p*_ *=* 10. This sensitivity assessment verified that our final enrolled cohort (N = 13) remained statistically sufficient to support the rank-based analysis without modifying the study’s original design or sample size determination. Considering the risk of drop-out during the experimental sessions, 15 soccer players volunteered to participate in the study (mean ± SD: age, 22.6 ± 2.03 years; stature, 1.78 ± 0.05 m; body mass, 74.7 ± 10.07 kg; BMI, 23.38 ± 3.02 kg·m^−2^). Eligibility criteria included at least five years experience of soccer training and participation in a regular training program comprising three sessions per week. Players were excluded if they reported any chronic disease or injury before enrolment. Throughout the experimental period, participants were instructed to avoid anti-inflammatory medications and vitamin supplementation and to refrain from strenuous lower-limb exercise for 24 h before familiarization and until completion of all assessments. None of the participants had previously undergone CWI or FR recovery interventions, and the sample included players from different playing positions. Following a comprehensive explanation of the study procedures, anticipated benefits, and potential risks, written informed consent was obtained from all participants. The study was conducted in accordance with the Declaration of Helsinki and was approved by the local research ethics committee (CPP-SUD: N° 0410/2022). The participant flow diagram is provided in Supplementary Figure S1.

### 2.2. Study Design

This randomized crossover trial was conducted in accordance with the CONSORT recommendations for randomized crossover studies [34] (Supplementary Table S1). The study was designed to compare the acute effects of 15 min CWI, FR, and passive recovery performed between two 5mSRTs on physical performance, biochemical responses, and perceived recovery-related outcomes, assessed at several time points relative to exercise.

One week before the experimental sessions, participants completed a familiarization visit during which the study procedures were explained and anthropometric measurements were obtained. During each experimental trial, participants performed two 5mSRTs separated by a 15 min recovery period. Although the 5mSRT does not fully reproduce the physiological demands of a competitive soccer match, its repeated accelerations, decelerations, and changes in direction reflect important soccer-specific movement demands [32].

To ensure balanced condition sequences and limit potential order effects, the order of the experimental conditions was established using a structured block randomization procedure with rank-based allocation. Accordingly, a random number was generated for each participant using the RAND() function in Microsoft Excel 2019 (Redmond, WA, USA), after which participants were ranked in ascending order. Recovery conditions were then assigned according to this ranking in blocks of five participants for passive recovery, CWI, and FR.

For each 5mSRT, the best distance (BD), total distance (TD), fatigue index (FI), and percentage decrement (PD) were determined. Rating of perceived exertion (RPE) was assessed immediately after each 5mSRT and again following the recovery intervention.

**Figure 1:**
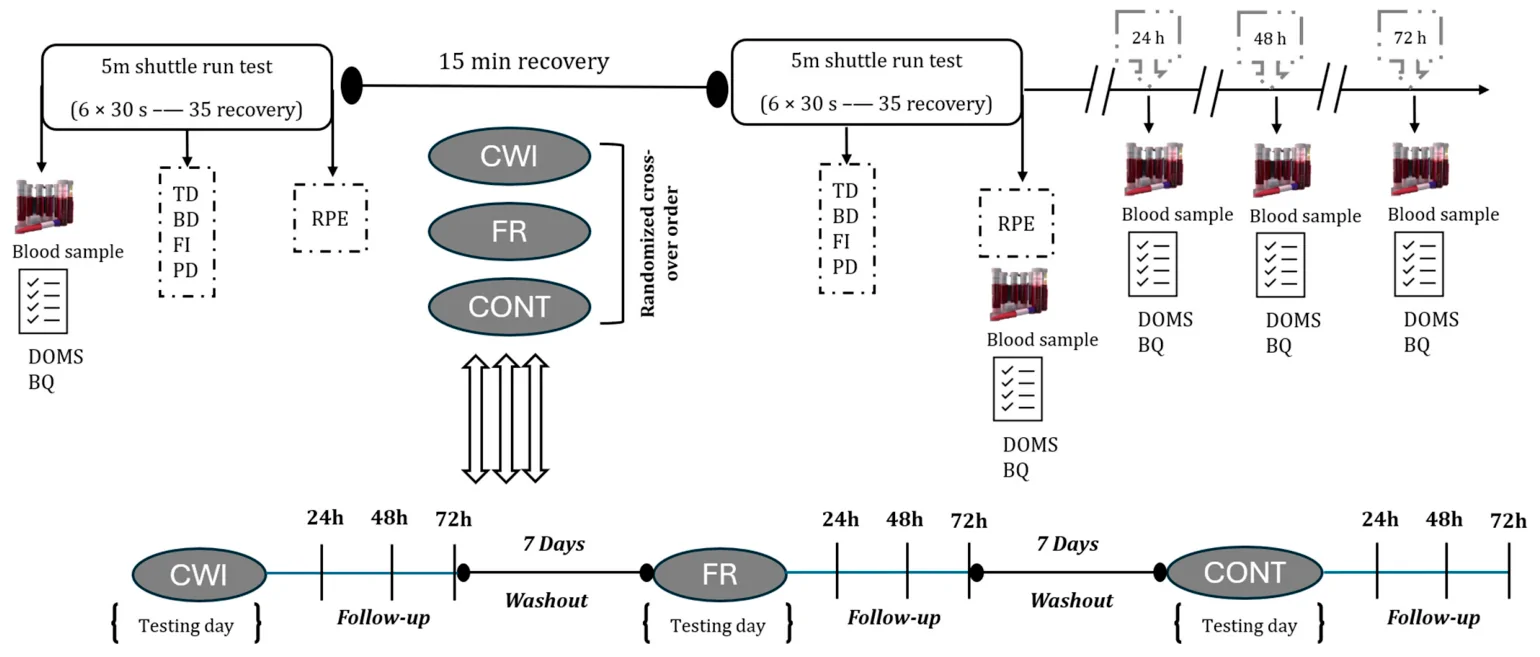
Schematic representation of the study design. FR: foam rolling, CWI: cold water immersion, CONT: control; TD: total distance, BD: best distance, FI: fatigue index; PD: performance decrement; RPE: rating of perceived exertion, DOMS: delayed onset muscle soreness, BQ: belief questionnaire.

During the 15 min recovery period, players were randomly allocated to either CWI, FR, or passive rest (i.e., control condition (CONT)). For each recovery condition, blood biomarkers of muscle damage [i.e., CK, lactate dehydrogenase (LDH), aspartate transaminase (AST)], inflammation [i.e., C-reactive protein (CRP)], delayed onset muscle soreness (DOMS) and belief questionnaire (BQ) were determined before the first 5mSRT and at different time points around the second 5mSRT (i.e., immediately after, 24 h, 48 h and 72 h) to ascertain the time-course of recovery of all variables. Moreover, to assure sufficient recovery and minimize potential carry-over effects, a 7-day washout period was implemented between the conditions (Figure 1). The washout period was counted since the last follow-up day (post-72 h follow-up). The sessions were conducted at the same time of day (09 h 00–11 h 00) to reduce the effect of diurnal variations in the performance.

#### Recovery Interventions

- CWI recovery: Participants immersed the lower body in cold water for 15 min, with water temperature maintained below 15 °C by adding ice when necessary. Temperature was continuously monitored using a liquid-in-glass thermometer (digital thermometer, 212–130, RS Products, Fort Worth, TX, USA). This protocol was consistent with recommendations suggesting immersion at 10–15 °C for 10–15 min to promote intramuscular cooling while preserving participant tolerance [19,35]. Previous evidence also indicates that comparable cooling responses may be obtained when the overall thermal load, defined by the combination of exposure duration and water temperature, is equivalent, with a 1:1 time–temperature approach, such as 15 min at 15 °C, commonly recommended [36]. During immersion, participants remained seated with the trunk and thighs positioned at approximately 90°, while the water level reached the superior iliac crest to ensure complete immersion of the lower limbs [21].
- FR recovery: Participants used a high-density, hollow-core foam roller with a grid-textured surface (diameter: 14 cm; length: 33 cm). Five exercises were performed targeting muscle groups commonly involved in soccer performance (i.e., quadriceps, hamstrings, adductors, gastrocnemius, gluteals). For each exercise, participants positioned the foam roller at the distal portion of the target muscle and applied as much body mass as comfortably tolerable. Rolling was then performed smoothly back and forth at a standardized cadence (∼one cycle per second) [24]. The complete FR protocol lasted 15 min. Each muscle group was treated bilaterally using two 35 s bouts per leg, separated by 10 s of rest [24].
- Passive recovery: Participants remained seated on a bench for 15 min to match the duration of the CWI and FR conditions. During this period, participants were instructed to refrain from any additional recovery activity.

### 2.3. Measures

#### 2.3.1. 5 m Shuttle Run Test (5mSRT)

The 5mSRT comprised six maximal 30 s shuttle-running bouts, each separated by 35 s of recovery. During each bout, players performed repeated all-out shuttle runs over progressively increasing distances of 5, 10, 15, and 20 m, continuing in the same manner thereafter [37]. Before the test, participants completed a standardized 15 min warm-up consisting of 10 min of low-intensity jogging, dynamic stretching and two submaximal shuttle runs to ensure adequate preparation. The distance covered during each 30 s bout was recorded, and the following performance indices were calculated:

- Best distance (BD) (m): the greatest distance covered during any single 30 s shuttle bout;
- Total distance (TD) (m): the cumulative (total) distance covered across all six shuttle bouts;
- Fatigue index (FI): calculates the drop-off in performance from the best to the worst performance and is calculated as follows.

Fatigue index (FI) (%):

(1) $$FI\left(\%\right)=\frac{\left[\frac{\mathrm{shuttle}1+\mathrm{shuttle}2}{2}-\frac{\mathrm{shuttle}5+\mathrm{shuttle}6}{2}\right]}{\frac{\mathrm{shuttle}1+\mathrm{shuttle}2}{2}}\times 100$$

Percentage decrement (PD) (%):

(2) $$PD\left(\%\right)=\frac{\left[\left(BD\times \mathrm{number}\mathrm{of}\mathrm{sprints}\right)-TD\right]}{\left(BD\times \mathrm{number}\mathrm{of}\mathrm{sprints}\right)}\times 100$$

#### 2.3.2. Ratings of Perceived Exertion (RPE)

RPE was assessed using the CR-10 Borg scale [38], ranging from 0 (nothing at all) to 10 (extremely strong), with intermediate verbal descriptors indicating increasing levels of perceived exertion.

#### 2.3.3. Delayed Onset Muscle Soreness (DOMS)

A 10 cm visual analog scale, anchored from 0 cm (“no pain”) to 10 cm (“extremely painful”), was used to assess perceived muscle soreness [39].

#### 2.3.4. Belief Questionnaire (BQ)

Participants rated the perceived effectiveness of each recovery modality using a six-point Likert scale ranging from 0 (“not effective at all”) to 5 (“extremely effective”) [40].

#### 2.3.5. Blood Samples

A 10 mL venous blood sample was obtained from a forearm vein and collected into heparinized tubes for the assessment of muscle damage biomarkers (LDH, CK and AST) and the inflammatory marker CRP. Immediately after collection, samples were placed in an ice bath and centrifuged (2500 rpm for 10 min). To reduce inter-assay variation, all samples were processed within the same analytical run. LDH, CK, and AST concentrations were measured using a kinetic assay at 340 nm, whereas CRP was determined by enzyme-linked immunosorbent assay (ELISA). All measurements were performed in duplicate in the same laboratory, with control serum analyzed simultaneously.

### 2.4. Statistical Analyses

Statistical analyses were conducted using SPSS version 27.0 (IBM Corp., Armonk, NY, USA). The Shapiro–Wilk test was used to assess data normality, Levene’s test to examine homogeneity of variances, and Mauchly’s test to evaluate sphericity. When the sphericity assumption was violated, Greenhouse–Geisser corrections were applied as required for TD.

Normally distributed variables (TD and RPE) are presented as mean ± SD, whereas non-normally distributed variables (ΔTD, BD, FI, PD, CK, LDH, CRP, AST, DOMS, and BQ) are reported as median and interquartile range (IQR). Parametric outcomes were analyzed using two-way repeated-measures ANOVA followed by Bonferroni-adjusted post hoc comparisons. Non-parametric outcomes were analyzed using the Friedman test followed by Wilcoxon signed-rank post hoc comparisons, with Holm–Bonferroni adjustment for multiple testing.

For parametric comparisons, effect sizes were expressed as Cohen’s d and interpreted as trivial (<0.20), small (0.20–<0.50), moderate (0.50–<0.80), large (0.80–<1.20), and very large (≥1.20) [41]. Corresponding 95% confidence intervals for between-condition differences (95% CIdiff) were also calculated. For non-parametric comparisons, effect sizes were calculated as r = Z/√N and interpreted as small (0.10–<0.30), moderate (0.30–<0.50), or large (≥0.50) [42]. Results were considered statistically significant when *p* < 0.05.

## 3. Results

### 3.1. The 5mSRT

#### 3.1.1. Total Distance

**Figure 2:**
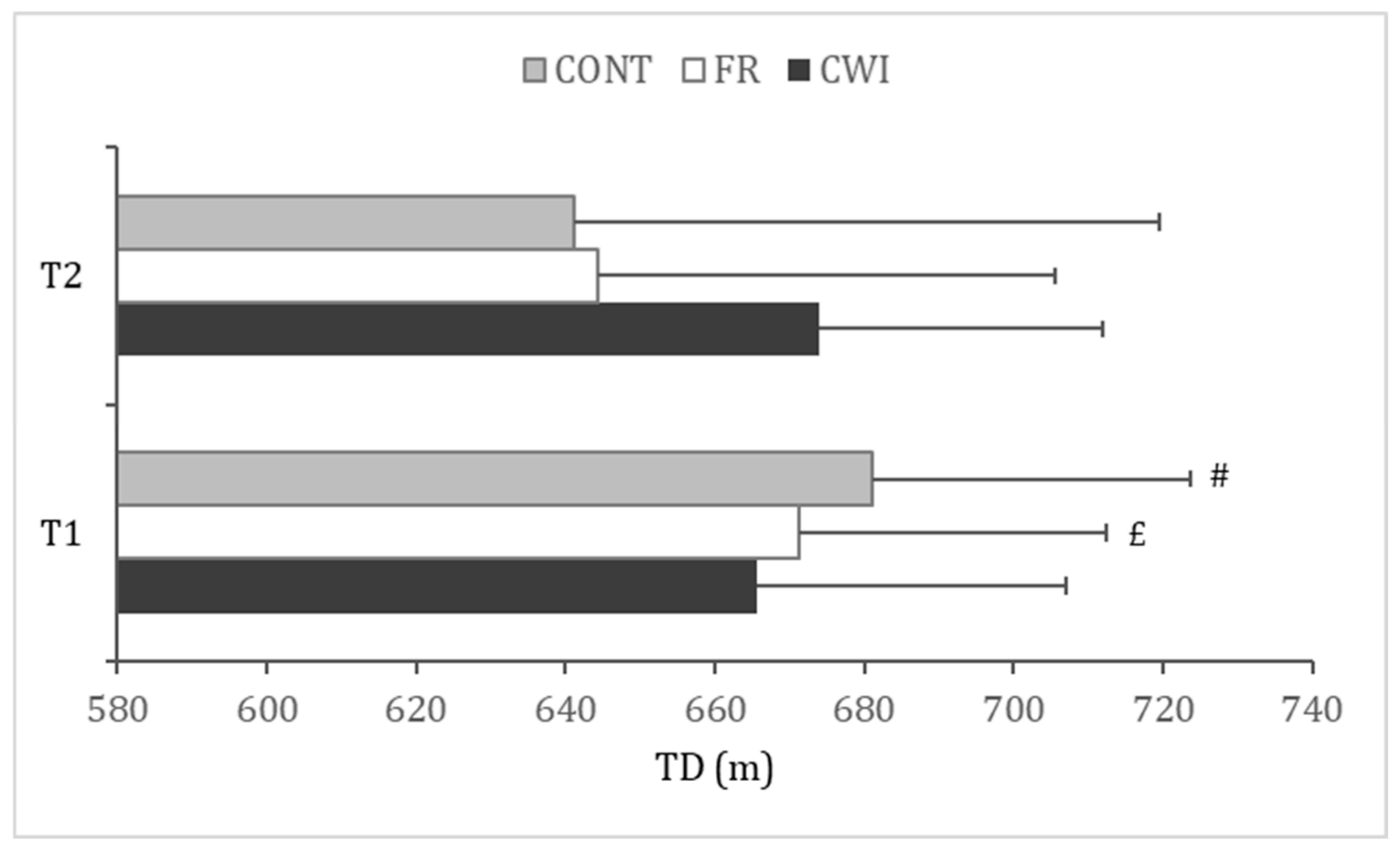
Variation in total distance (TD) between the first and second 5mSRT across conditions (control (CONT), foam rolling (FR), and cold water immersion (CWI)) and time. #: higher than CONT at T2; £: higher than FR at T2; T1: before recovery; T2: after recovery. Values are presented as mean (SD).

There was no significant effect of condition on TD (F_(2,13)_ = 0.40; η_p_^2^ = 0.06; *p =* 0.68). However, there was a significant effect of time (F_(1,14)_ = 10.37; η_p_^2^ = 0.43; *p =* 0.006), with greater TD during the first than the second 5mSRT (95%CI_diff_ = 6.51 to 32.46; d = 0.37; *p =* 0.006). Moreover, there was a significant condition × time interaction (F_(1.30,18.29)_ = 4.13; η_p_^2^ = 0.23; *p =* 0.048). The post hoc analysis revealed a significant decrease in TD from T1 to T2 after CONT (95%CI_diff_ = −74.20 to −5.93; d = −0.65; *p =* 0.025) and FR (95%CI_diff_ = −50.28 to −3.43; d = −0.64; *p =* 0.028), with no significant change after CWI (*p =* 0.21) (Figure 2).

#### 3.1.2. Best Distance

**Figure 3:**
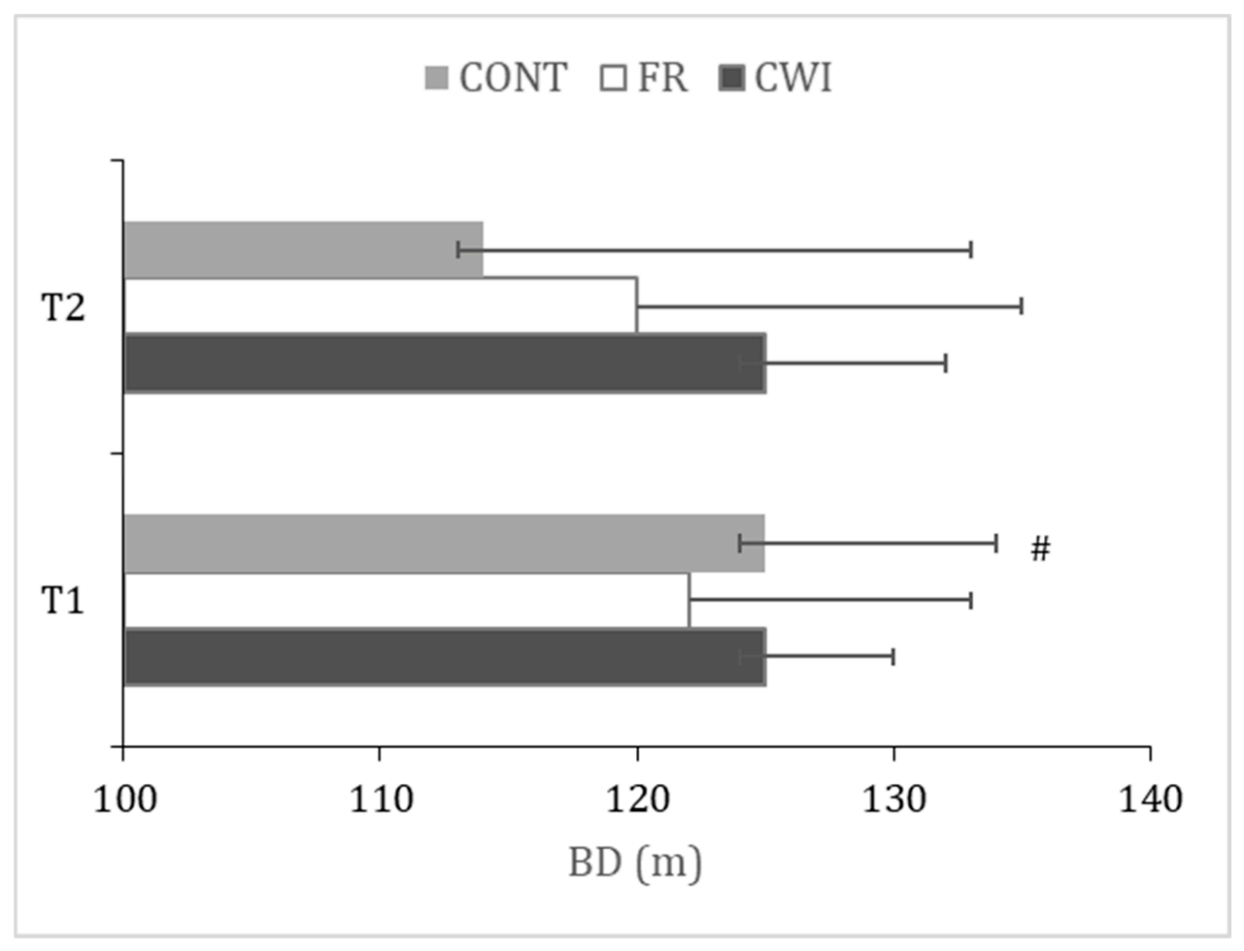
Variation in best distance (BD) between the first and second 5mSRT across conditions (control (CONT), foam rolling (FR), and cold water immersion (CWI)) and time. #: higher than CONT at T2. T1: before recovery; T2: after recovery. Values are presented as MED/IQR.

There was a significant effect of condition on BD (χ^2^(5) = 13.79; *p =* 0.017). The Wilcoxon signed-rank test showed that, when comparing post-recovery to before, the CONT condition elicited shorter BD (z = −2.94; r = 0.76; *p =* 0.03) (Figure 3).

#### 3.1.3. Fatigue Index and Performance Decrement

**Figure 4:**
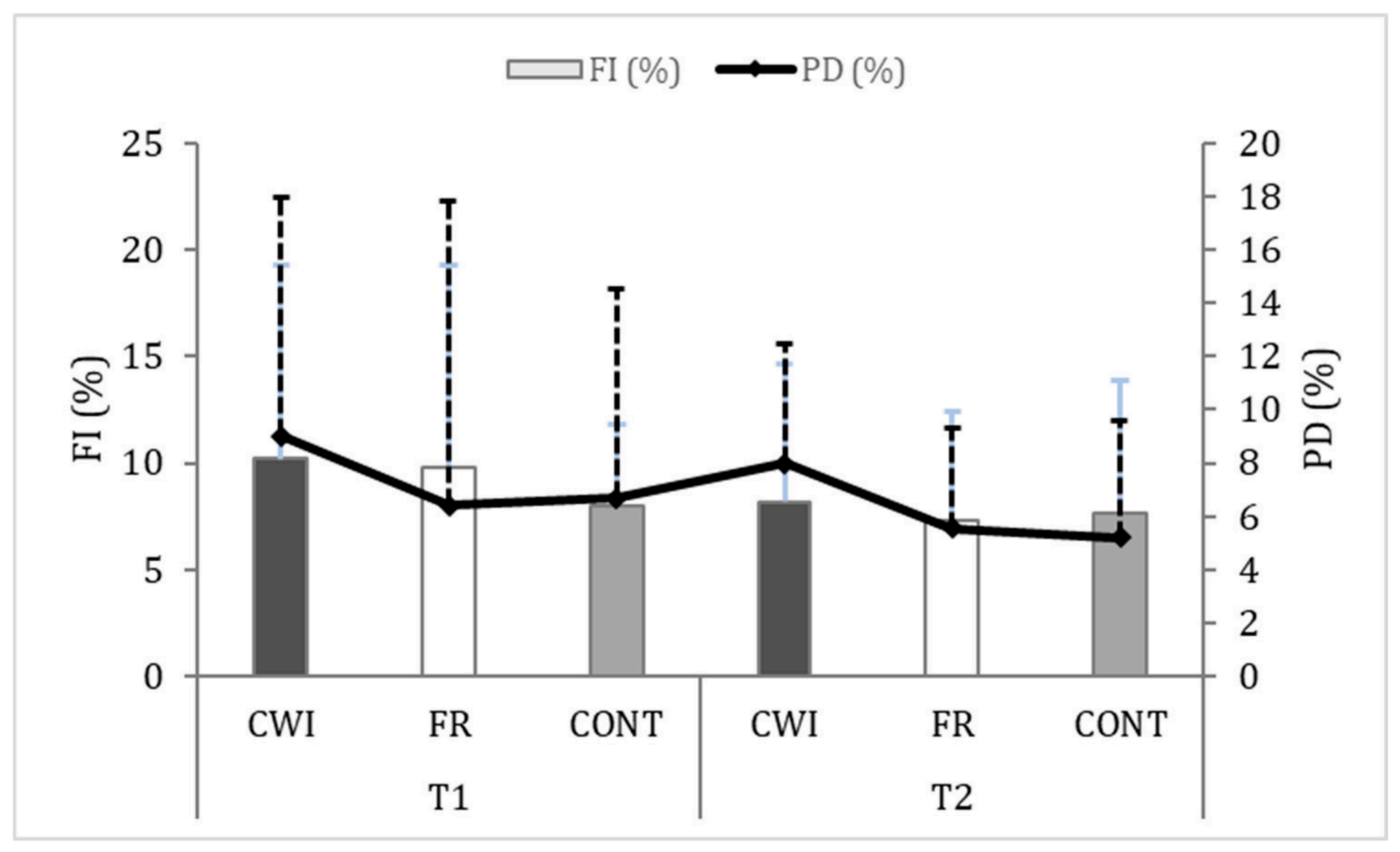
Variation in fatigue index (FI) and performance decrement (PD) between the first and second 5mSRT across conditions (control (CONT), foam rolling (FR), and cold water immersion (CWI)) and time. T1: before recovery; T2: after recovery. Values are presented as MED/IQR; blue lines indicates e the standard deviation of the FI; The dashed lines indicates the standard deviation of the performance decrement.

There was no significant effect of condition on FI (χ^2^(5) = 3.60; *p =* 0.61) and PD (χ^2^(5) = 3.76; *p =* 0.59) (Figure 4).

### 3.2. Biochemical Parameters

Variations in muscle damage and inflammation biomarkers across conditions and time are presented in Table 1.

#### 3.2.1. Creatine Kinase (CK)

CK showed a significant effect of condition (χ^2^(14) = 152.20, *p* < 0.001), with significantly lower CK concentrations following CWI vs. CONT at 48 h (z = −3.12, r = −0.80, *p =* 0.003).

#### 3.2.2. Lactate Dehydrogenase (LDH)

A significant effect of condition was observed for LDH (χ^2^(14) = 114.73, *p* < 0.001), with significantly lower LDH concentrations following CWI vs. CONT at 48 h (z = −3.29, r = −0.85, *p =* 0.01) and 72 h (z = −3.12, r = −0.81, *p =* 0.02).

#### 3.2.3. Aspartate Aminotransferase (AST)

AST demonstrated a significant effect of condition (χ^2^(14) = 99.09, *p* < 0.001), with significantly lower AST concentrations following FR vs. CONT at 48 h (z = −3.35, r = −0.86, *p =* 0.01). No significant between-condition differences were observed at the remaining time points.

#### 3.2.4. C-Reactive Protein (CRP)

CRP also showed a significant effect of condition (χ^2^(14) = 40.78, *p* < 0.001), with no significant differences between recovery conditions at any individual time point.

### 3.3. Rating of Perceived Exertion (RPE)

**Figure 5:**
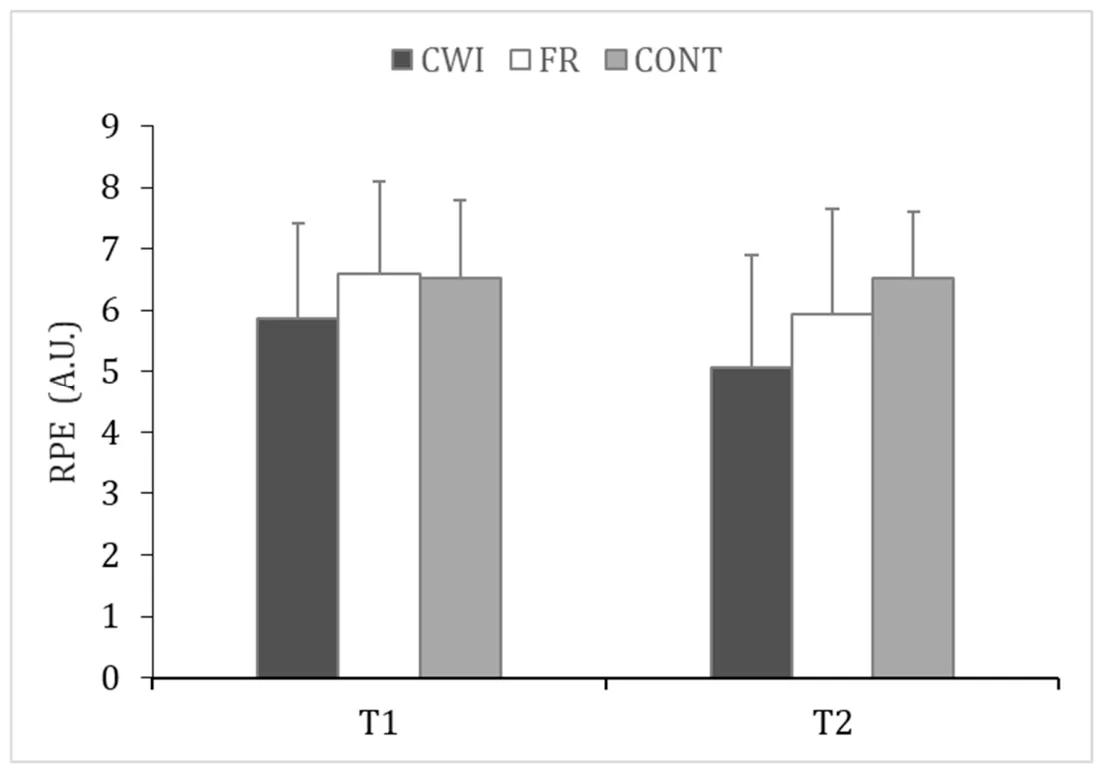
Variation in rating of perceived exertion (RPE) across conditions (control (CONT), foam rolling (FR), and cold water immersion (CWI)) and time. T1: before recovery; T2: after recovery; AU: arbitrary units. Values are presented as mean ± SD.

There was no significant effect of condition (F_(2,13)_ = 2.87; η_p_^2^ = 0.31; *p =* 0.93), timing (F_(1,14)_ = 1.66; η_p_^2^ = 0.11; *p* = 0.22) or interaction (F_(2,13)_ = 1.66; η_p_^2^ = 0.20; *p =* 0.23) on RPE (Figure 5).

### 3.4. Delayed Onset of Muscle Soreness (DOMS)

**Figure 6:**
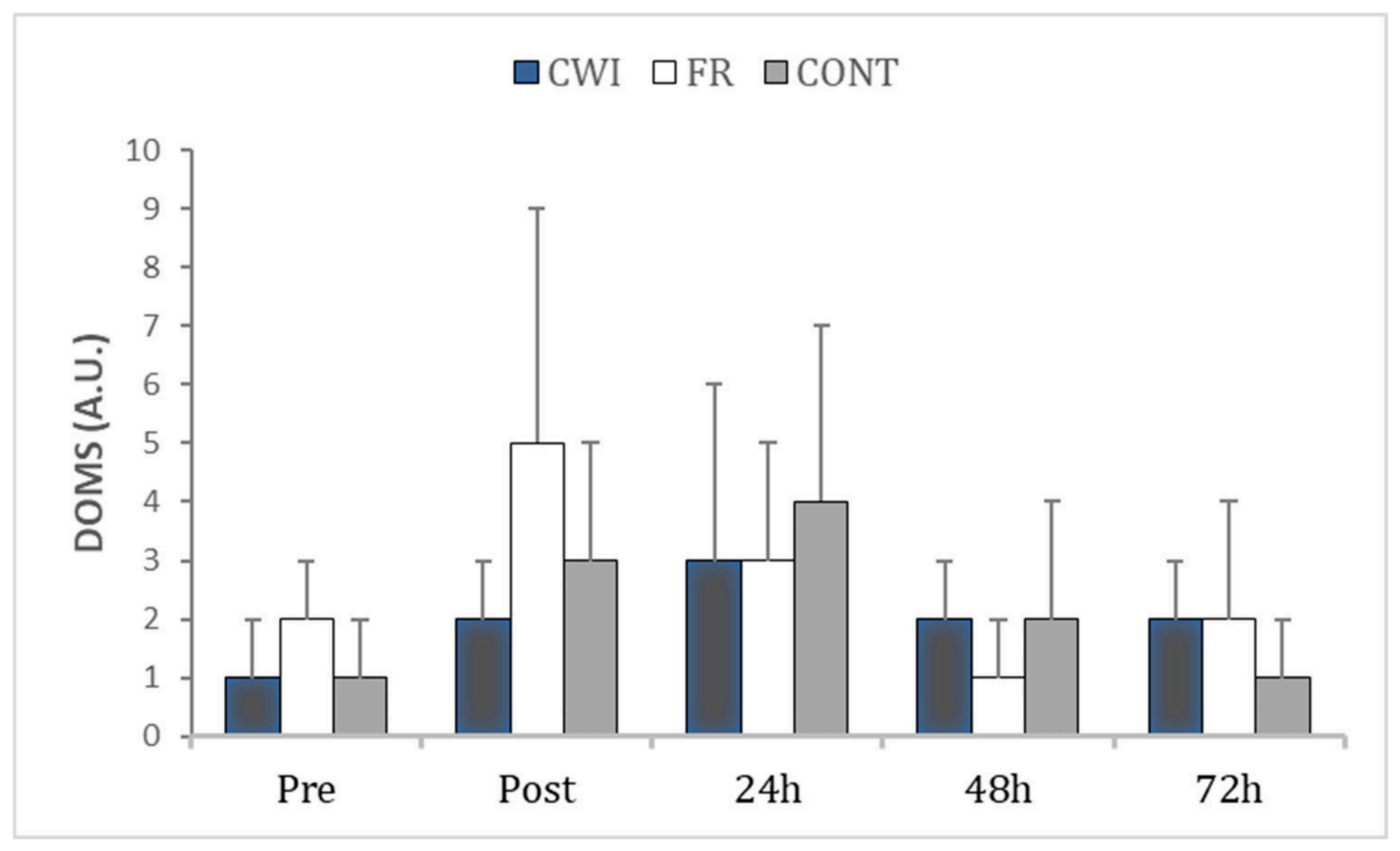
Variation in delayed onset muscle soreness (DOMS) scores across conditions and time. T1: before recovery; T2: after recovery. FR: foam rolling; AU: arbitrary units. Values are presented as MED–IQR.

There was a significant effect of condition on DOMS (χ^2^(14) = 69.48, *p* < 0.001), with no significant differences detected between conditions at any individual time point (Figure 6).

### 3.5. Belief Questionnaire (BQ)

**Figure 7:**
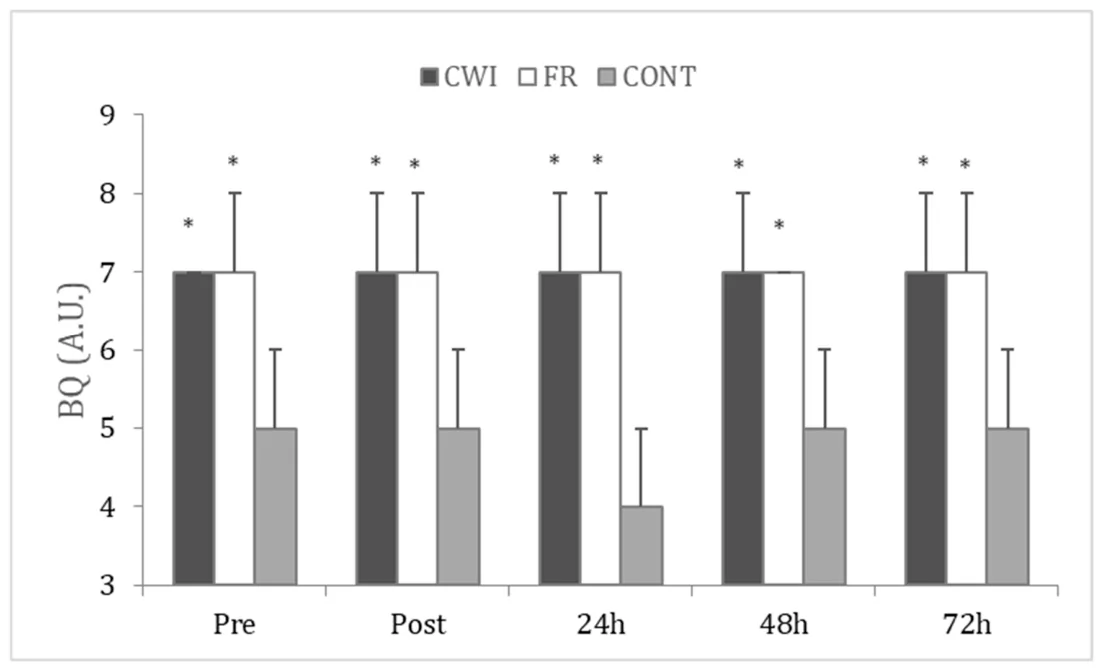
Variation in belief questionnaire (BQ) scores across conditions (control (CONT), foam rolling (FR), and cold water immersion (CWI)) and time. *: Higher than control (CONT); CWI: cold water immersion; AU: arbitrary units. Values are presented as MED/IQR.

A significant effect of condition was observed for BQ (χ^2^(14) = 154.61, *p* < 0.001). Post hoc analysis showed that, before testing, BQ scores were significantly higher in CWI (z = −3.49, r = 0.90, *p =* 0.003) and FR (z = −3.34, r = 0.86, *p =* 0.007) compared with CONT. Immediately after testing, both CWI (z = −3.46, r = 0.86, *p =* 0.003) and FR (z = −3.22, r = 0.83, *p =* 0.008) elicited significantly higher scores than CONT. Similarly, BQ scores remained significantly higher than CONT after 24 h for CWI (z = −3.34, r = 0.86, *p =* 0.007) and FR (z = −3.46, r = 0.89, *p =* 0.006), after 48 h for CWI (z = −3.45, r = 0.89, *p =* 0.006) and FR (z = −3.36, r = 0.87, *p =* 0.007), and after 72 h for CWI (z = −3.46, r = 0.89, *p =* 0.006) and FR (z = −3.47, r = 0.90, *p =* 0.003) (Figure 7).

## 4. Discussion

The current study compared the effects of CWI, FR, and passive recovery applied between two 5mSRTs. The findings indicate that CWI preserved repeated-running performance, as TD remained unchanged following recovery, whereas significant declines were observed following passive recovery and FR. In addition, CWI attenuated selected biomarkers of muscle damage, including CK and LDH, compared with passive recovery. In contrast, no between-condition differences were observed for FI, PD, RPE, DOMS, or CRP. Although both CWI and FR elicited higher recovery belief scores than passive recovery, FR demonstrated only limited physiological effects, with a reduction in AST observed at 48 h.

The capacity of CWI to preserve absolute performance volume was evidenced by the finding that TD remained statistically unchanged post-intervention, whereas significant performance decrements occurred following both passive recovery and FR. Additionally, BD exhibited a significant within-condition decrement exclusively under the passive recovery framework. This aligns with previous crossover investigations demonstrating that CWI can buffer acute physical performance drops following high-intensity intermittent strain [7,21,43,44]. Relative performance preservation under CWI might be related to the potential of CWI to acutely blunt both central and peripheral components of neuromuscular fatigue [21]. The physiological responses induced by CWI may influence performance during the second 5mSRT through several interacting mechanisms. Peripheral vasoconstriction and reductions in tissue temperature and metabolic activity may contribute to attenuating inflammatory responses [43]. At the same time, acute cooling may transiently reduce nerve conduction velocity and motor-unit firing rate, potentially impairing neuromuscular performance [18]. During the subsequent reperfusion phase, increased vasodilation may facilitate blood flow redistribution [45], while improved tissue reoxygenation and clearance of metabolic by-products may further support recovery [18]. However, direct between-condition comparisons for the second 5mSRT performance did not demonstrate universal superiority. The complete absence of condition effects for the FI and PD further underscores this limitation. The lack of variance in these relative fatigue indices indicates that while CWI may help stabilize absolute capacity and buffer covered distance drops, it does not fundamentally alter the underlying rate or magnitude of performance decay during repeated maximal shuttle efforts. Consequently, supporting the outcome-dependence theory [29], claims regarding the efficacy of CWI must be strictly confined to the relative maintenance of absolute distance metrics under specific conditions, rather than a broad-spectrum optimization of physical capacity.

The observed preservation of TD performance could be explained by the improved time-course recovery at the biochemical level [30]. In the present study, CWI drove an acute mitigation of muscle damage markers, yielding lower systemic concentrations of CK and LDH relative to passive recovery, supporting previous evidence showing reductions in muscle damage indices following recovery [13,17,20,46]. Because parameters such as intramuscular tissue temperature, localized microvascular blood flow, and cell membrane permeability were not directly quantified in the present study, the exact physical pathways underlying this stabilization remain strictly hypothetical. It is possible that the hydrostatic pressure and thermal vasoconstriction native to immersion may have restricted the localized space available for edema formation, thereby slowing the efflux of intracellular enzymes into the bloodstream [18,47]. In contrast to the broad-spectrum enzyme suppression observed under CWI at 48 h, FR demonstrated a highly selective, delayed biochemical utility by reducing AST concentrations. This time-dependent divergence aligns with observations by Pearcey, Bradbury-Squires, Kawamoto, Drinkwater, Behm and Button [24], suggesting that mechanical soft tissue stimulation operates on a distinct physiological window. By mechanically manipulating the soft tissues, FR likely alleviates local cross-bridge restrictions and increases deep microcirculation [48]. This mechanical pumping action potentially accelerated the clearance of localized interstitial fluid accumulations, allowing systemic AST clearance to begin normalizing by 48 h. Importantly, the protective biochemical effects identified in this cohort were strictly confined to these localized indices of mechanical cell leakage (CK, LDH, and AST). Contrary to previous studies suggesting a generalized anti-inflammatory effect for cryotherapy [49], no between-condition differences were observed for CRP concentrations at any time point. This absolute lack of divergence in systemic inflammation matches recent investigations indicating that while localized mechanical or thermal therapies can successfully buffer structural cell membrane leakage, they do not blunt acute-phase systemic immune or hepatic responses such as CRP or interleukin-6 [50]. This indicates that while acute recovery modalities can successfully stabilize localized structural cell leakage, a single session of CWI or FR is ultimately insufficient to alter the overarching systemic inflammatory cascade following repeated maximal shuttle efforts.

In the present study, the findings did not demonstrate a clear advantage of either recovery strategy in reducing perceived muscle soreness following repeated maximal shuttle-running exercise. Although previous investigations have reported acute analgesic effects of FR [8,24,51] and CWI [10,21,52], the magnitude and persistence of these responses may vary according to the exercise model, recovery strategy, intervention characteristics, and population studied [1,19]. Klich et al. [53] similarly reported an immediate improvement in pressure pain threshold following CWI, although this analgesic response was not consistently maintained during the subsequent recovery period. Such variability may partly explain the absence of significant differences between recovery conditions in the present study. Therefore, a single acute exposure to CWI or FR may have limited influence on the complex physiological processes contributing to post-exercise muscle soreness during prolonged recovery [54]. The descriptive trends observed here should nevertheless be interpreted cautiously, and studies involving larger samples are needed to clarify the effects of these recovery strategies on post-exercise muscle soreness in soccer players.

Within our cohort, the belief questionnaire revealed significantly higher scores for both CWI and FR compared to passive recovery before and after the second 5mSRT, with no significant differences observed between the two active modalities. This strong psychological alignment suggests that the preservation of TD under the CWI condition was likely driven by a potent combination of early psychological expectancy and physiological stabilization pathways (CK and LDH). The observed increase in players’ belief in these active protocols could have contributed to performance preservation through psychological or placebo effects [40]. The widespread adoption of CWI among soccer players is largely attributed to its perceived ability to reduce muscle soreness and feelings of fatigue, thereby facilitating recovery of performance [30]. These perceived benefits may partly reflect immediate improvements in subjective recovery and affective state [18]. Cold exposure may also stimulate sympathetic activity and increase circulating catecholamine concentrations, potentially enhancing alertness and feelings of vitality and, consequently, contributing to a positive expectancy or placebo-related response [55]. Ultimately, these expectancies and acute physiological effects of CWI likely preserve muscle contractile capacity, maintaining higher physical output during repeated maximal efforts [19,47] and accelerating the restoration of normal physiological function [56]. However, the fact that TD was maintained only under CWI, and not under FR, despite both interventions sharing similar elevated belief scores, exposes the physiological limits of pure psychological expectancy. While a positive expectancy placebo can alter subjective sensations of fatigue and modulate pain thresholds [57], it cannot unilaterally overcome acute structural cellular damage. Therefore, the high psychological belief associated with FR was ultimately insufficient to buffer physical performance declines.

A major strength of this study is its comprehensive assessment of biochemical, perceptual, and performance-related recovery outcomes, allowing a clear comparison between recovery strategies. Nevertheless, several methodological limitations should be acknowledged. First, the study examined the acute response to a single recovery intervention. Future research should investigate the cumulative impact of repeated CWI exposure across a competitive season, particularly during congested schedules involving several matches per week, to determine whether this strategy may contribute to injury-risk reduction and sustained performance. Second, while biochemical, perceptual, and performance measures were included, additional mechanistic assessments (e.g., neuromuscular activation via electromyography, tissue oxygenation via near-infrared spectroscopy) were not directly measured, meaning that localized vascular and neuro-electrical explanations remain strictly theoretical. Third, the small sample included only male soccer players, limiting the generalizability of the findings. Larger studies including female players are needed to explore possible sex-related differences in recovery responses. Fourth, the use of a simulated shuttle-running protocol rather than actual match play limits the ecological validity of the findings, as the protocol did not reproduce the technical, tactical, psychological, and contextual demands of competitive soccer. Therefore, the results should be extrapolated to match-play recovery with caution. Finally, although participant expectations were assessed using the BQ, blinding participants to CWI and FR was not feasible, which represents an inherent limitation of research involving physical recovery interventions.

## 5. Practical Applications

Our findings suggest that CWI may be considered when the objective is to acutely preserve absolute running volume and reduce selected circulating markers associated with muscle damage. Within the boundaries of this simulated paradigm, CWI demonstrated potential utility for short-term workload maintenance during condensed schedules or between repeated high-intensity training sessions. Based on the experimental protocol, sport science staff may consider 15 min of lower-body immersion at approximately 15 °C as a feasible strategy to help maintain total distance capacity during subsequent bouts of intermittent effort. However, because these outcomes were derived from a controlled laboratory simulation rather than real-world game data, direct extrapolation to competitive match performance should be made cautiously. Alternatively, FR represents a practical, low-cost, and easily accessible recovery option, particularly when water immersion facilities are unavailable. Our results suggest that FR operates on a distinct, time-dependent timeline, requiring a longer recovery window to exert meaningful effects on delayed biochemical recovery markers (e.g., AST). Therefore, FR may be more appropriately positioned as a complementary component of post-training or post-match recovery, rather than as a primary intervention when rapid between-effort performance restoration is required.

Because no differences were observed between conditions for subjective muscle soreness, perceived exertion, or systemic inflammation (CRP), practitioners should select these modalities based on highly specific physical or biochemical targets rather than assuming a generalized, uniform attenuation of match-induced fatigue.

## 6. Conclusions

CWI performed at 15 °C for 15 min may represent a useful recovery strategy for soccer players following repeated high-intensity intermittent exercise. CWI demonstrated greater efficacy than passive recovery specifically in attenuating TD decrements and reducing post-exercise circulating CK and LDH concentrations following repeated maximal shuttle efforts in soccer players. However, these recovery benefits were parameter-dependent, as no significant condition effects were observed for FI, PD, RPE, DOMS, or systemic inflammatory markers (CRP). Furthermore, FR demonstrated localized utility during delayed recovery, as evidenced by reduced AST concentrations at 48 h. These findings suggest that CWI may help maintain total running volume and mitigate acute muscle damage during congested competitive schedules. Nevertheless, practitioners should select recovery modalities based on specific target outcomes, recognizing that neither CWI nor FR provide a uniform benefit across all performance, perceptual, and biochemical parameters.

## Figures and Tables

**Table 1:** Variations in muscle damage and inflammation biomarkers across conditions and time. Values are presented as MED/IQR.

Variable	Condition	Time				
		Pre	Post	24 h	48 h	72 h
CK (U/L)	CWI	248/81	495/226	475/249	361/115 ^§^	237/72
	FR	241/101	630/291	720/444	420/295	289.00/187
	CONT	237/102	562/272	552/433	415/126	280/63
LDH (U/L)	CWI	180/40	243/172	304/132	261/114 ^§^	202/29 ^¥^
	FR	194/71	252/319	385/110	302/105	234/35
	CONT	180/57	390/255	491/194	425/163	300/116
CRP (mg/L)	CWI	0.99/0.91	1.25/0.77	2.20/2.89	2.87/3.50	1.55/1.48
	FR	1/0.65	1.74/0.62	3.50/4.46	2.80/5.53	1.85/2.74
	CONT	1.13/0.50	1.96/1.18	4.93/4.92	3.81/6.36	2.65/3.80
AST(U/L)	CWI	29/8	61/30	39/24	37/19	28/11
	FR	28/6	62/42	51/18	38/18 ^§^	31/12
	CONT	25/7	63/38	57/12	54/10	38/13

CONT: control; CWI: cold water immersion; FR: foam rolling; AST: aspartate aminotransferase; CK: creatine kinase; CRP: C-reactive protein; LDH: lactate dehydrogenase; ^§^: lower vs. CONT after 48 h; ^¥^: lower vs. CONT after 72 h.

## Data Availability

Data is contained within the article.

## Supplementary Materials

The following supporting information can be downloaded at https://www.mdpi.com/article/10.3390/sports14080328/s1: Figure S1: Participant flow diagram; Table S1: The Consolidated Standards of Reporting Trials Checklist of the study.

## Abbreviations

The following abbreviations are used in this manuscript:

BQ	Belief questionnaire
DOMS	Delayed onset muscle soreness
FI	Fatigue index
PD	Percentage decrement
5mSRT	5 m shuttle run test
TD	Total distance
BD	Best distance
RPE	Rating of perceived exertion
CWI	Cold water immersion
FR	Foam rolling
CK	Creatine Kinase
LDH	Stanford Sleepiness Scale
CRP	C-reactive protein
AST	Aspartate aminotransferase
